# Alanine Aminotransferase and Bilirubin Dynamic Evolution Pattern as a Novel Model for the Prediction of Acute Liver Failure in Drug-Induced Liver Injury

**DOI:** 10.3389/fphar.2022.934467

**Published:** 2022-07-22

**Authors:** Ruiyuan Yang, Kexin Li, Cailun Zou, Aileen Wee, Jimin Liu, Liwei Liu, Min Li, Ting Wu, Yu Wang, Zikun Ma, Yan Wang, Jingyi Liu, Ang Huang, Ying Sun, Binxia Chang, Qingsheng Liang, Jidong Jia, Zhengsheng Zou, Xinyan Zhao

**Affiliations:** ^1^ Liver Research Center, Beijing Friendship Hospital, Capital Medical University, National Clinical Research Center for Digestive Diseases, Beijing, China; ^2^ Department of Pathology, Yong Loo Lin School of Medicine, National University of Singapore, Singapore, Singapore; ^3^ Department of Pathology and Molecular Medicine, Faculty of Health Sciences, McMaster University, Hamilton, ON, Canada; ^4^ Fourth Department of Liver Disease (Difficult and Complicated Liver Diseases and Artificial Liver Center), Beijing You’an Hospital, Capital Medical University, Beijing, China; ^5^ Clinical Epidemiology and Evidence Base Medicine Unit, Beijing Friendship Hospital, Capital Medical University, Beijing, China; ^6^ Department of Critical Liver Diseases, Liver Research Center, Beijing Friendship Hospital, Capital Medical University, National Clinical Research Center for Digestive Diseases, Beijing, China; ^7^ Senior Department of Hepatology, The Fifth Medical Center of PLA General Hospital, Beijing, China

**Keywords:** drug toxicity, predictive model, dynamic evolution pattern, clinical characteristic, clinical outcome

## Abstract

**Aims:** To develop, optimize, and validate a novel model using alanine aminotransferase (ALT) and total bilirubin (TB) dynamic evolution patterns in predicting acute liver failure (ALF) in drug-induced liver injury (DILI) patients.

**Methods:** The demographics, clinical data, liver biopsy, and outcomes of DILI patients were collected from two hospitals. According to the dynamic evolution of ALT and TB after DILI onset, the enrolled patients were divided into ALT-mono-peak, TB-mono-peak, double-overlap-peak, and double-separate-peak (DSP) patterns and compared. Logistic regression was used to develop this predictive model in both discovery and validation cohorts.

**Results:** The proportion of ALF was significantly higher in patients with the DSP pattern than in the ALT-mono-peak pattern and DOP pattern (10.0 vs. 0.0% vs. 1.8%,*p* < 0.05). The area under receiver operating characteristic curve (AUROC) of the DSP pattern model was 0.720 (95% CI: 0.682–0.756) in the discovery cohort and 0.828 (95% CI: 0.788–0.864) in the validation cohort in predicting ALF, being further improved by combining with international normalized ratio (INR) and alkaline phosphatase (ALP) (AUROC in the discovery cohort: 0.899; validation cohort: 0.958). Histopathologically, patients with the DSP pattern exhibited a predominantly cholestatic hepatitis pattern (75.0%, *p* < 0.05) with a higher degree of necrosis (29.2%, *p* = 0.084).

**Conclusion:** DILI patients with the DSP pattern are more likely to progress to ALF. The predictive potency of the model for ALF can be improved by incorporating INR and ALP. This novel model allows for better identification of high-risk DILI patients, enabling timely measures to be instituted for better outcome.

## Introduction

There have been dramatic changes in recent decades in the spectrum of liver diseases with a far-reaching impact on healthcare systems worldwide. The majority of chronic hepatitis C patients can be cured (Dennis et al., 2021), and chronic hepatitis B can be effectively controlled (European Association for the Study of the Liver, 2017). Drug-induced liver injury (DILI) has gradually emerged as a relatively common clinical liver disease with significant derangement of liver biochemical tests. Epidemiological data suggest that the annual incidence of DILI is 2.7–13.9 per 100,000 in Europe and North America (Vega et al., 2017; Sgro et al., 2002); it is even higher in the Asia-Pacific region (Suk et al., 2012; Shen et al., 2019) with an annual incidence of 13.9–23.8 per 100,000.

DILI is the most common cause of acute liver failure (ALF) in Europe and North America (Khandelwal et al., 2011; Reuben et al., 2016; Stravitz and Lee, 2019), and the mortality associated with DILI-induced ALF is as high as 80% if liver transplantation had not been performed (Stravitz and Lee, 2019). A recent prospective study from the Drug-Induced Liver Injury Network (DILIN) found that 10% of patients died or required liver transplantation within 2 years of DILI onset, in 80% of which DILI played a major or contributory role. (Hayashi et al., 2017).

Early identification of DILI-induced ALF is critical in clinical practice so that timely measures can be adopted to improve the final outcome. Various predictive models of ALF have been established. Hyman Zimmerman’s model (Hy’s Law), the most impactful, was used for early prediction of ALF during drug development and in clinical settings. It was validated by the Spanish DILI registry (Andrade et al., 2005) Swedish Adverse Drug Reactions Advisory Committee (SADRAC) database (Björnsson and Olsson, 2005) and the US DILIN (Chalasani et al., 2008; Andrade and Robles-Díaz, 2020). Subsequently, Spanish scholars updated Hy’s law and proposed a novel independent prognostic algorithm (named Robles-Diaz Model in our study) for DILI-induced ALF to achieve a better balance between sensitivity and specificity (Robles-Diaz et al., 2014; Lo Re et al., 2015). However, these models are based on specific values of liver biochemical parameter(s) at a single time point (onset/peak), and their predictive capability can certainly be improved. Whether the dynamic evolution patterns of ALT and TB in patients with DILI can be used as a new model to predict DILI-induced ALF has not been studied yet.

In this study, a novel model based on the dynamic evolution of ALT and TB in predicting DILI-induced ALF was established, optimized, and validated. The significance was to assist in early and accurate identification of high-risk DILI patients for timely intervention to improve clinical outcome.

## Patients and Methods

### Study Subjects

The study population was divided into the discovery cohort and the validation cohort. From January 2016 to December 2018, the medical records of patients with DILI were retrieved as the discovery cohort at the Senior Department of Hepatology, the Fifth Medical Center of PLA General Hospital, Beijing, China. Additionally, patients between January 2013 and December 2020 at the Liver Research Center, Beijing Friendship Hospital, Capital Medical University, Beijing, China, were included in the external validation cohort.

1.1 Inclusion criteria: 1) Age ≥ 18 years; 2) the chronological sequence between drug and liver injury was clear; 3) the Roussel–Uclaf Causality Assessment Method (RUCAM) score is ≥6.
1.2 Exclusion criteria: 1) Acute viral hepatitis A to E, Epstein-Barr virus or cytomegalovirus infection, autoimmune liver diseases (autoimmune hepatitis, primary biliary cholangitis, and primary sclerosing cholangitis), non-alcoholic steatohepatitis, alcoholic liver disease, hereditary and metabolic liver diseases, biliary obstruction, and ischemic hepatitis; 2) systemic infections (such as sepsis); 3) organ transplantation; and 4) malignant tumor of the liver, bile duct, or pancreas.

### Study Methods

#### Retrieval of Onset Data and Hospitalization Data

Demographic, clinical, and laboratory data at the onset and hospital admission were retrieved, including blood routine, liver biochemical tests, lipid profiles, international normalized ratio (INR), viral hepatitis, and autoimmune markers. The dynamic evolution of ALT and TB during the course of the disease was recorded, and the corresponding patterns were established.

### Clinical Classification, Causality, and Severity of Drug-Induced Liver Injury

Clinical classification of DILI was based on the Council for International Organizations of Medical Sciences (CIOMS) criteria: hepatocellular injury type: R ≥ 5, cholestatic: R ≤ 2, and mixed: 2 < R < 5 (Aithal et al., 2011).

The causality of the drug to liver injury was assessed using the Roussel Ucalaf causality assessment (RUCAM) (Danan and Teschke, 2015).

Severity of cases was graded as mild, moderate, severe, acute liver failure, and fatal according to the Chinese 2015 DILI guidelines (Yu et al., 2017).

### Criteria for Prediction Models of Drug-Induced Liver Injury-Induced Acute Liver Failure

The current prediction models are as follows:

(i) Hy’s law: ALT or AST >3×ULN and TB > 2×ULN, ALP <2×ULN (Temple, 2006);
(ii) New Hy’s law (nHy’s law): TB > 2×ULN, nR ≥ 5 [nR value defined as (measured highest ALT or AST/their ULN)/(measured ALP/ALP ULN)] (Robles-Diaz et al., 2014); and
(iii) Robles-Diaz Model (Robles-Diaz et al., 2014): AST >17.3×ULN and TB > 6.6×ULN, or AST ≤17.3×ULN, but AST/ALT >1.5 (Björnsson and Olsson, 2005).

### Definition of Alanine Aminotransferase-Total Bilirubin Dynamic Evolution Patterns

**FIGURE 1:**
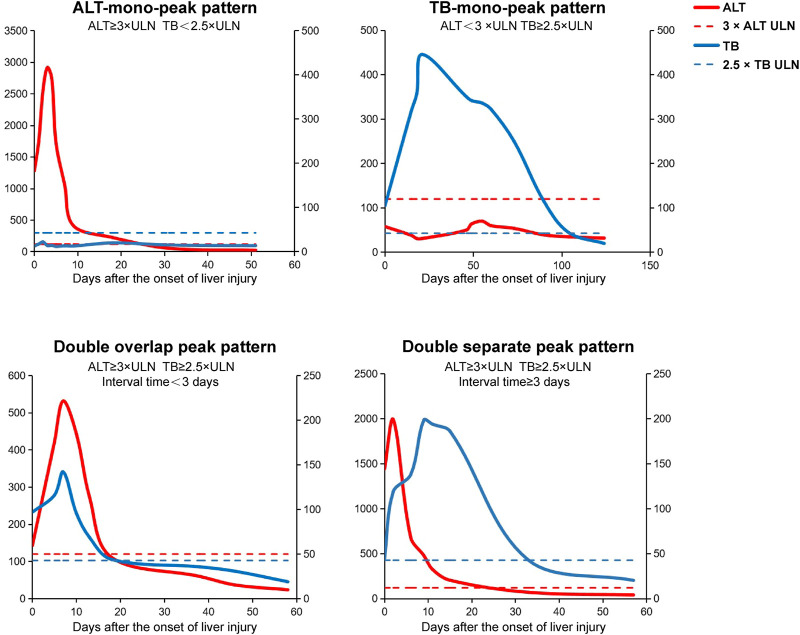
Schematic diagram of the four different dynamic evolution patterns of ALT and TB in DILI. The ALT-TB dynamic evolution pattern is defined as the following four patterns: (1) ALT-mono-peak pattern, ALT ≥ 3×ULN, and TB < 2.5×ULN; (2) TB-mono-peak pattern, ALT < 3×ULN and TB ≥ 2.5×ULN; (3) ALT and TB double overlap peak pattern, ALT ≥ 3×ULN and TB ≥ 2.5×ULN, and the time interval between ALT and TB peak <3 days; (4) ALT and TB double separate peak pattern, ALT ≥ 3×ULN and TB ≥ 2.5×ULN, and the time interval between ALT and TB peaks ≥3 days. Abbreviations: DILI, drug-induced liver injury; ALT, alanine aminotransferase; TB, total bilirubin.

In order to establish a new prediction model for predicting ALF after the onset of DILI, the ALT-TB dynamic evolution patterns are defined as the following four patterns (Figure 1): 1) ALT-mono-peak pattern: ALT ≥3×ULN and TB < 2.5×ULN; 2) TB-mono-peak pattern: ALT <3×ULN and TB ≥ 2.5×ULN; 3) ALT and TB double overlap peak (DOP) pattern: ALT ≥3×ULN and TB ≥ 2.5×ULN, with the time interval between ALT and TB peaks being <3 days; and 4) ALT and TB double separate peak (DSP) pattern: ALT ≥3×ULN and TB ≥ 2.5×ULN, with the time interval between ALT and TB peaks being ≥3 days.

### Assessment of Liver Pathology

The liver biopsies were stained with hematoxylin and eosin (H&E), reticulin, Masson trichrome, periodic acid-Schiff with diastase (PAS-D), cytokeratin 7 (CK7), and CK19. Liver biopsies of enrolled patients (if any) were reviewed and classified by a clinical liver pathologist (XYZ) according to the pathological classification of DILI by Kleiner et al. (2014) and Wang et al. (2019).

### Follow-Up and Definition of Clinical Outcomes for Drug-Induced Liver Injury

Follow-up within 1 year of DILI onset was achieved by means of a hospital management system (HIS) query, telephone consultation, and post-discharge laboratory test records. The clinical outcomes included ALF or death/liver transplantation.

The definition of ALF is according to the American Association for the Study of Liver Diseases (AASLD) guideline for the management of ALF (Polson and Lee, 2005). Liver-related deaths include (Hayashi et al., 2017) 1) DILI directly causing death and 2) aggravation of DILI or induction of another fatal disease (e.g., sepsis, multiorgan failure, and others).

### Statistical Analysis

All data were analyzed using R version 3.3.3 and SPSS software (version 21.0; IBM Corp., Armonk, NY). Differences between groups were analyzed by ANOVA analysis for normally distributed variables, the Kruskal–Wallis H-test for non-normally distributed continuous variables, and the Chi-square test for categorical data. Mann–Whitney *U* tests were performed for multiple comparisons. Bilateral *p* < 0.05 was regarded as statistical difference.

The receiver operating characteristic (ROC) curves were used to analyze the prognostic performance of the previously published models and our novel model, and the area under ROC curve (AUROC) of the different models was compared by the Delong method. Logistic regression and the bootstrap method were used to develop and validate the optimized model in discovery and validation cohorts. In logistic regression, variables with *p* < 0.05 in the univariate analysis were screened as input variables and independent variables were screened using a likelihood ratio-based forward method to establish a logistic regression model.

This study has been approved by the Medical Ethics Committee of Beijing Friendship Hospital, Capital Medical University (Approval No.: 2020-P2-071-01), and the informed consent form has been waived.

## Results

### Demographic and Clinical Characteristics According to Alanine Aminotransferase-Total Bilirubin Dynamic Evolution Patterns

**FIGURE 2:**
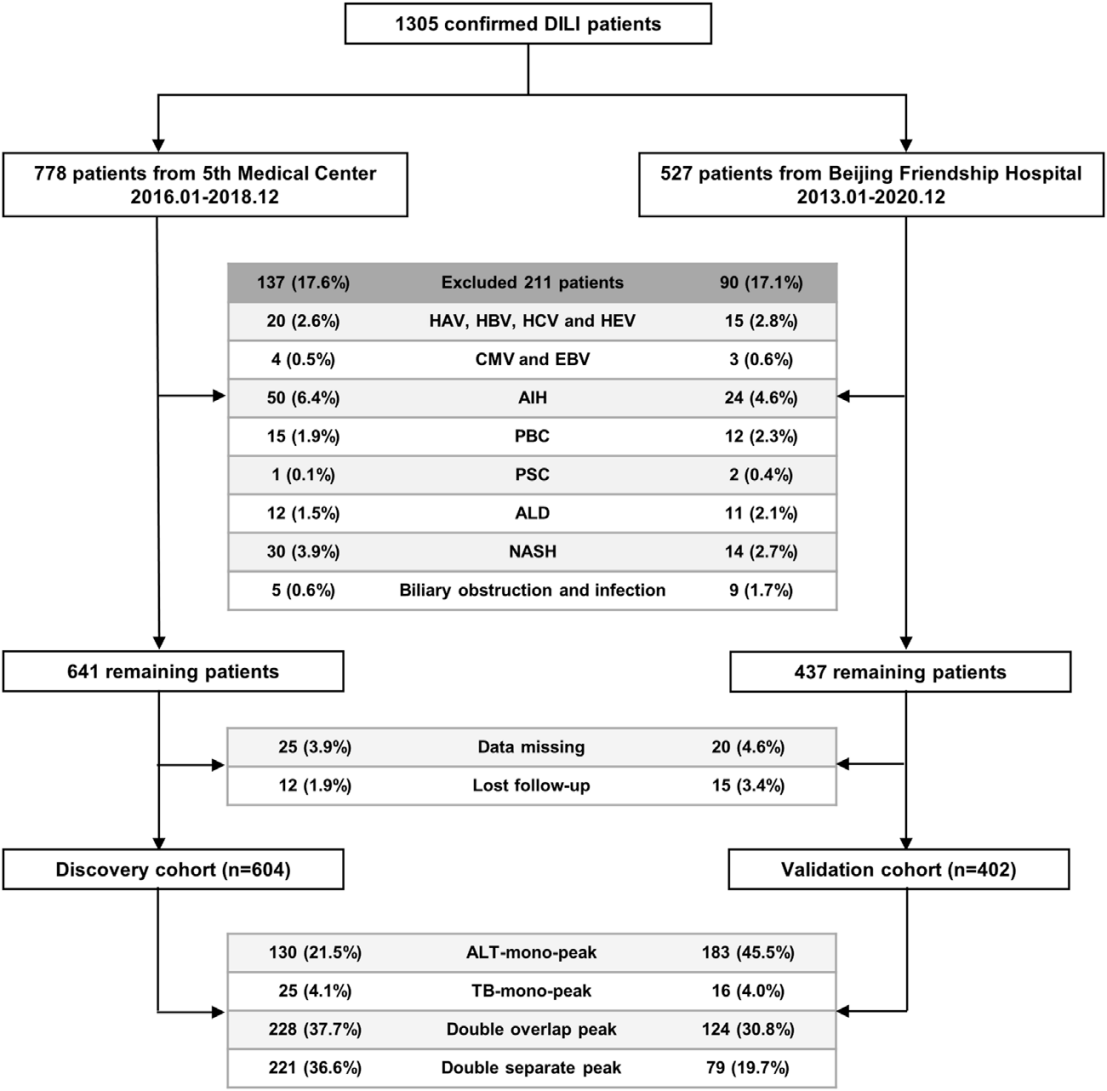
Flowchart of the enrolled patients with DILI in the discovery and validation cohorts. Abbreviations: DILI, drug-induced liver injury; HAV, hepatitis A virus; HBV, hepatitis B virus; HCV, hepatitis C virus; HEV, hepatitis E virus; CMV, cytomegalovirus; EBV, Epstein-Barr virus; AIH, autoimmune hepatitis; PBC, primary biliary cholangitis; PSC, primary sclerosing cholangitis; ALD, alcoholic liver disease; NASH, non-alcoholic steatohepatitis; ALT, alanine aminotransferase; TB, Total bilirubin.

Patients diagnosed with DILI at the Fifth Medical Center of PLA General Hospital were enrolled in the discovery cohort (604 cases) from January 2016 to December 2018, and patients diagnosed with DILI at the Beijing Friendship Hospital, Capital Medical University, were enrolled in the external validation cohort (402 cases) from January 2013 to December 2020 (Figure 2).

In the discovery cohort, 372 out of 604 cases (61.6%) were female, with a median age of 49 years. The common clinical symptoms were jaundice (78.6%), fatigue (74.2%), and poor appetite (70.5%). Hepatocellular injury was predominant (80.0%). There was no significant difference in age, sex, latency, and BMI among the four dynamic evolution patterns (*p* > 0.05) (Table 1). The median hospitalization time was significantly longer (23.0 days) in patients with the DSP pattern.

**TABLE 1:** Comparison of the demographic data and liver biochemical parameters among the four ALT-TB dynamic evolution patterns in DILI.

Discovery cohort	Total (*n* = 604)	ALT-mono-peak (*n* = 130)	TB-mono-peak (*n* = 25)	ALT and TB double overlap peak (*n* = 228)	ALT and TB double separate peak (*n* = 221)	*p* value
Female (n, %)	372 (61.6)	90 (69.2)	14 (56.0)	137 (60.1)	131 (59.3)	0.235
Age (years)	49.0 (40.0, 57.0)	50.0 (39.0, 58.0)	54.0 (49.00, 62.0)	48.5 (40.0, 56.0)	48.0 (40.0, 57.0)	0.111
BMI (kg/m^2^)	23.3 (21.3, 25.4)	23.9 (22.1, 25.4)	22.1 (19.0, 24.9)	23.1 (21.4, 25.3)	23.3 (21.2, 25.4)	0.209
Latency (days)	27.0 (10.0, 50.0)	30.0 (12.0, 60.0)	30.0 (14.0, 90.0)	30.0 (10.0, 60.0)	20.0 (10.0, 40.0)	0.404
*R*-value at onset	17.0 (6.9, 31.2)	15.2 (9.4, 29.5)	0.9 (0.5, 1.8)	20.1 (9.5, 32.7)	16.4 (5.7, 32.1)	**<0.001** ^◆**▲▼**^
Selected liver biochemical tests at DILI onset						
ALT (U/L)	809.5 (321.8, 1262.8)	570.0 (324.0, 954.0)	67.0 (41.0, 85.0)	950.5 (506.0, 1307.5)	843.0 (338.0, 1422.0)	**<0.001** ^◆●**★▲▼**^
AST (U/L)	546.5 (203.8, 931.0)	352.0 (187.0, 692.0)	60.0 (47.0, 86.0)	668.5 (335.0, 956.5)	605.0 (223.0, 1026.0)	**<0.001** ^◆●**★▲▼**^
ALP (U/L)	166.0 (127.0, 226.0)	131.0 (94.0, 180.0)	235.0 (196.0, 348.0)	168.5 (130.5, 215.0)	178.0 (140.0, 246.0)	**<0.001** ^◆●**★▲▼**^
GGT (U/L)	179.0 (100.3, 311.8)	137.0 (72.0, 250.0)	114.0 (62.0, 431.0)	183.0 (117.5, 328.5)	206.0 (118.0, 308.0)	**<0.001** ^●**★**^
TB (μmol/L)	113.5 (40.5, 200.1)	19.9 (14.7, 26.5)	131.0 (114.5, 311.9)	140.7 (88.9, 232.0)	150.0 (90.2, 207.5)	**<0.001** ^◆●**★**^
DB (μmol/L)	79.8 (22.6, 148.5)	8.0 (5.5, 13.8)	104.9 (65.8, 254.0)	100.1 (60.2, 175.1)	105.7 (64.0, 153.3)	**<0.001** ^◆●**★**^
ALB (g/L)	36.0 (32.0, 38.0)	38.0 (36.0, 41.0)	33.0 (30.0, 36.0)	36.0 (32.0, 38.0)	34.0 (31.0, 37.0)	**<0.001** ^◆●**★▲✝**^
CHE (KU/L)	5439.0 (4367.0, 6480.0)	6602.0 (5677.0, 7685.0)	4681.0 (2586.0, 5280.0)	5458.0 (4502.0, 6429.0)	4823.0 (3900.0, 5870.0)	**<0.001** ^◆●**★▲✝**^
TBA (μmol/L)	80.5 (15.0, 219.8)	8.0 (4.0, 15.0)	94.0 (33.0, 247.0)	62.0 (18.0, 179.5)	204.0 (115.0, 283.0)	**<0.001** ^◆●**★✝**^
Cr (μmol/L)	64.0 (52.0, 76.0)	61.0 (52.0, 73.3)	57.0 (47.0, 76.0)	63.0 (52.0, 75.0)	67.0 (55.0, 81.5)	**0.009** ^**★**^
CHOL (mmol/L)	3.9 (3.2, 4.8)	4.2 (3.5, 4.9)	4.2 (3.5, 4.9)	4.0 (3.4, 4.7)	3.6 (2.9, 4.7)	**0.003** ^**★**^
TG (mmol/L)	2.1 (1.4, 3.2)	1.2 (1.0, 1.6)	3.2 (2.5, 4.2)	2.0 (1.4, 2.7)	2.9 (2.2, 4.0)	**<0.001** ^◆●**★▲✝**^
INR	1.0 (0.9, 1.1)	1.0 (0.9, 1.0)	1.0 (0.9, 1.0)	1.0 (0.9, 1.1)	1.0 (1.0, 1.2)	**<0.001** ^●**★▼✝**^
Selected liver biochemical tests at their peak time						
ALT (U/L)	829.5 (365.5, 1276.3)	603.0 (386.0, 1080.0)	81.0 (61.0, 97.0)	961.5 (518.0, 1334.0)	855.0 (405.0, 1455.0)	**<0.001** ^◆●**▲▼**^
AST (U/L)	583.5 (247.3, 965.8)	378.0 (222.0, 703.0)	82.0 (58.0, 109.0)	675.5 (372.0, 963.5)	657.0 (272.0, 1095.0)	**<0.001** ^◆●**★▲▼**^
ALP (U/L)	185.5 (139.3, 244.0)	142.0 (106.0, 202.0)	294.0 (210.0, 401.0)	180.0 (138.0, 232.5)	206.0 (157.0, 281.0)	**<0.001** ^◆●**★▲▼✝**^
GGT (U/L)	202.0 (117.0, 365.0)	170.0 (79.0, 311.0)	133.0 (92.0, 494.0)	206.5 (129.0, 386.5)	230.0 (137.0, 353.0)	**<0.001** ^●**★**^
TB (μmol/L)	145.9 (53.9, 281.6)	21.2 (15.7, 29.3)	307.8 (145.1, 386.8)	142.4 (89.5, 235.35)	260.9 (159.1, 361.5)	**<0.001** ^◆●**★✝**^

Abbreviations: DILI, drug-induced liver injury; BMI, body mass index; ALT, alanine aminotransferase; AST, aspartate aminotransferase; ALP, alkaline phosphatase; GGT, glutamyltransferase; TB, total bilirubin; DB, direct bilirubin; ALB, albumin; CHE, cholinesterase; TBA, total bile acid; Cr, creatinine; CHOL, cholesterol; TG, triglycerides; INR, International normalized ratio. Data were presented as median (quartile). ^◆^There is a statistical difference between ALT-mono-peak and TB-mono-peak. ^●^There is a statistical difference between ALT-mono-peak and double overlap peak. ^★^There is a statistical difference between ALT-mono-peak and double separate peak. ^▲^There is a statistical difference between TB-mono-peak and double overlap peak. ^▼^There is a statistical difference between TB-mono-peak and double separate peak. ^**✝**^There is a statistical difference between double overlap peak and double separate peak.

At DILI onset, ALT, AST, ALP, GGT, TB, DB, and INR were significantly higher in the two double-peak patterns than in the ALT-mono-peak pattern (*p* < 0.05) (Table 1). Furthermore, INR levels were significantly higher in the DSP pattern than in the DOP pattern. Total bile acid (TBA) levels were significantly higher in the DSP pattern than in the ALT-mono-peak pattern and DOP pattern (*p* < 0.05), and its median values were 20 times higher than the upper limit of normal. Albumin (ALB) and cholinesterase (CHE) levels were significantly lower in the DSP pattern than that in the ALT-mono-peak pattern and DOP pattern (*p* < 0.05). At the peak level of biochemical tests, TB and ALP were significantly higher in the DSP pattern than in the ALT-mono-peak pattern and DOP pattern (*p* < 0.05). The proportion of ALF was significantly higher in patients with the DSP pattern than in the ALT-mono-peak pattern and DOP pattern (10.0 vs. 0.0% vs. 1.8%, *p* < 0.05) (Table 2).

**TABLE 2:** Comparison of clinical classification, severity, and outcomes among the four ALT-TB dynamic evolution patterns in DILI.

Discovery cohort	Total (*n* = 604)	ALT-mono-peak (*n* = 130)	TB-mono-peak (*n* = 25)	ALT and TB double overlap peak (*n* = 228)	ALT and TB double separate peak (*n* = 221)	*p* value
Injury pattern, n (%)						**<0.001**
Hepatocellular	483 (80.0)	116 (89.2)	0 (0.0)	197 (86.4)	170 (76.9)	^◆★▲▼^
Cholestatic	43 (7.1)	2 (1.5)	20 (80.0)	9 (3.9)	12 (5.4)	^◆▲▼^
Mixed	78 (12.9)	12 (9.2)	5 (20.0)	22 (9.6)	39 (17.6)	
Culprit drug(s), n (%)						**0.017**
HDS	302 (50.0)	48 (36.9)	12 (48.0)	121 (53.1)	121 (53.1)	^●★^
Drugs	119 (19.7)	34 (26.2)	7 (28.0)	35 (15.4)	43 (19.5)	
HDS + Drugs	183 (30.3)	48 (36.9)	6 (24.0)	72 (31.6)	57 (25.8)	
Severity, n (%)						**<0.001**
Mild	155 (25.7)	130 (100.0)	2 (8.0)	0 (0.0)	23 (10.4)	^◆●★▲**✝**^
Moderate	79 (13.1)	0 (0.0)	3 (12.0)	52 (22.8)	24 (10.9)	^◆●★**✝**^
Severe	339 (56.1)	0 (0.0)	17 (68.0)	172 (75.4)	150 (67.9)	^◆●★^
ALF/Fatal	31 (5.1)	0 (0.0)	3 (12.0)	4 (1.8)	24 (10.9)	^◆★▲**✝**^
Outcomes, n (%)						
Acute liver failure	28 (4.6)	0 (0.0)	2 (8.0)	4 (1.8)	22 (10.0)	**<0.001** ^◆★**✝**^
Liver-related Death/LT	13 (2.2)	0 (0.0)	2 (8.0)	0 (0.0)	11 (5.0)	**<0.001** ^◆▲**✝**^
Duration of hospitalization (days), n (%)	15.0 (11.0–23.0)	11.0 (7.0–15.0)	15.0 (10.0–29.0)	13.5 (9.0–19.0)	23.0 (15.0–32.0)	**<0.001** ^◆●★**✝**^

Abbreviations: DILI, drug-induced liver injury; HDS, herbal and dietary supplements; ALT, alanine aminotransferase; TB, total bilirubin; ALF, acute liver failure; LT, liver transplantation. Data were presented as median (quartile). ^◆^There is a statistical difference between ALT-mono-peak and TB-mono-peak. ^●^There is a statistical difference between ALT-mono-peak and double overlap peak. ^★^There is a statistical difference between ALT-mono-peak and double separate peak. ^▲^There is a statistical difference between TB-mono-peak and double overlap peak. ^▼^There is a statistical difference between TB-mono-peak and double separate peak. ^**✝**^There is a statistical difference between double overlap peak and double separate peak.

### Comparison of Clinical Characteristics and Laboratory Data at Drug-Induced Liver Injury Onset Between the ALF/non-ALF Group and Drug-Induced Liver Injury With/Without Non-Alcoholic Fatty Liver Disease Group

The laboratory tests at DILI onset showed that INR, TB, and TBAs were significantly higher, while ALB was significantly lower in the ALF group than in the non-ALF group (*p* < 0.05) (Supplementary Table S4). No significant difference in ALT, AST, ALP, and GGT was found between the two groups (Supplementary Table S4). In the discovery cohort, 99 of 604 cases (16.3%) had underline NAFLD. No significant difference was found in the outcomes of ALF and liver-related death/LT between the two groups (Supplementary Table S5). There was no significant difference between the DILI with NAFLD and DILI without the NAFLD group except for BMI, GGT, and the proportion of females (Supplementary Table S5).

### Prediction of Acute Liver Failure According to Alanine Aminotransferase-Total Bilirubin Dynamic Evolution Patterns

As shown in Table 2, the two double-peak pattern groups had a significantly higher proportion of ALF than the two mono-peak pattern groups: 22 cases (10.0%) in the DSP and four (1.8%) in the DOP patterns but none in the ALT-mono-peak pattern (*p* < 0.001). The DSP pattern had the worst outcomes—22 patients (10.0%) developed ALF and 11 (5%) developed liver-related death.

**FIGURE 3:**
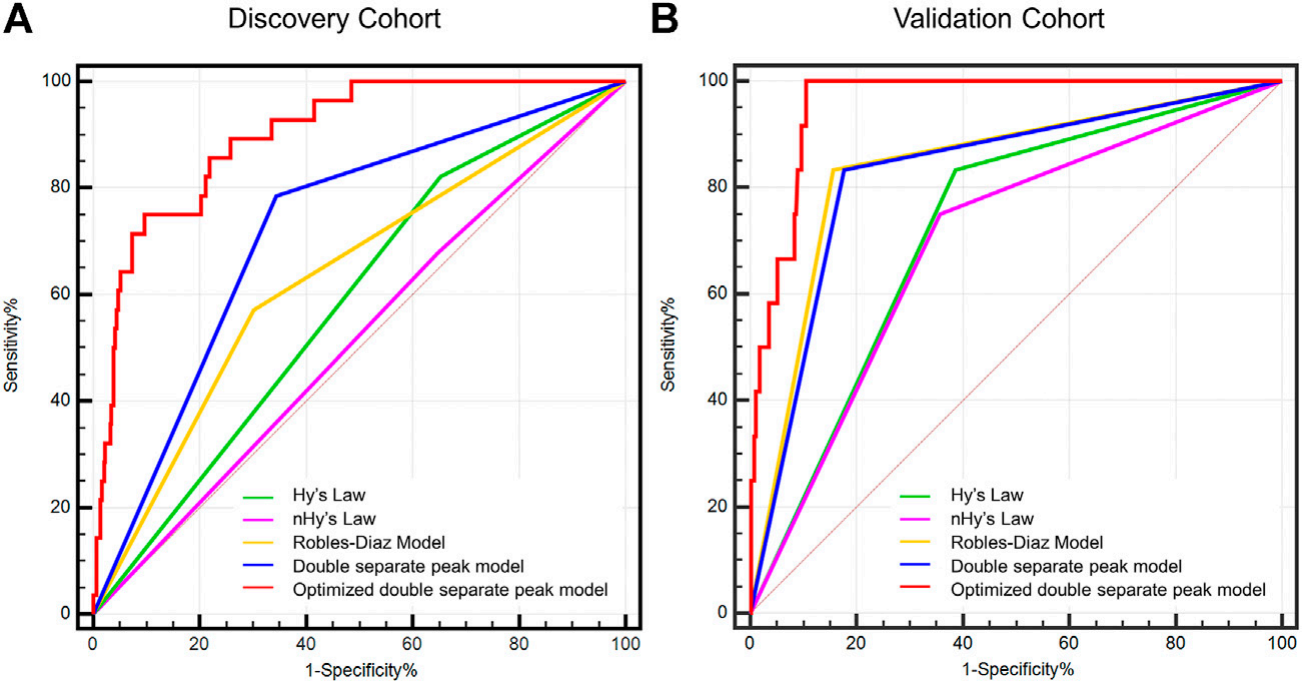
Potency comparison among different models in predicting DILI-induced ALF. The AUROC of the DSP model was significantly superior to that of Hy’s law and nHy’s law models and comparable with the Robles-Diaz Model. The prediction potency of ALF was further improved when incorporated with INR and ALP at DILI onset, which was significantly better than the three previous models with an AUROC for predicting DILI-induced ALF of 0.899 (95% CI: 0.87–0.921) in the discovery cohort and 0.958 (95% CI: 0.933–0.975) in the validation cohort, respectively [Figure **(A)** and **(B)**]. Abbreviations: AUROC, area under receiver operating characteristic; DSP, ALT, and TB double separate peak patterns; nHy’s Law, new Hy’s Law; DILI, drug-induced liver injury; ALT, alanine aminotransferase; TB, total bilirubin.

As a novel model for the prediction of ALF, the sensitivity and specificity of the DSP pattern were 78.6 and 65.5%, respectively. The AUROC of the DSP model was 0.720 (95% CI: 0.682–0.756), whereas the AUROCs of Hy’s law, nHy’s law, and Robles-Diaz Model were 0.515 (95% CI: 0.474–0.555), 0.583 (95% CI: 0.543–0.623), and 0.635 (95% CI: 0.595–0.673), respectively (Table 3). The AUROC of the DSP model was significantly superior to the Hy’s law and nHy’s law models (Figure 3) (*Z* = 3.386, *p* < 0.001 or *Z* = 2.757, *p* = 0.006), comparable with the Robles-Diaz Model (*Z* = 1.296, *p* = 0.195).

**TABLE 3:** Comparison of the double separate peak model with existing prediction models.

	Discovery cohort				Validation cohort	
	AUROC (95% CI)	*p* Value	Sensitivity (%)	Specificity (%)	AUROC (95% CI)	*p* Value
Hy’s Law	0.515 (0.474–0.555)	<0.001	67.86	35.07	0.723 (0.677–0.766)	<0.001
nHy’s Law	0.583 (0.543–0.623)	<0.001	82.14	34.55	0.696 (0.648–0.740)	<0.001
Robles-Diaz Model	0.635 (0.595–0.673)	<0.001	57.14	69.79	0.838 (0.799–0.873)	0.038
Double separate peak model	0.720 (0.682–0.756)	<0.001	78.57	65.45	0.828 (0.788–0.864)	0.034
Optimized double separate peak model	0.899 (0.872–0.922)	Ref	75.00	90.45	0.958 (0.933–0.975)	Ref

Abbreviations: AUROC, area under receiver operating characteristic; CI, confidence interval; nHy’s law, new Hy’s law. AUROC of Hy’s law, nHy’s law, Robles-Diaz Model, and ALT and TB double separate peak models were all compared with the optimized double separate peak model.

The AUROC of the DSP pattern in DILI with NAFLD patients was 0.859 (95% CI: 0.775–0.921), with a sensitivity of 100.0% and a specificity of 71.9%.

### Verification of Alanine Aminotransferase-Total Bilirubin Dynamic Evolution Patterns in the Prediction of Acute Liver Failure

We conducted both internal and external verification on the potency of the DSP model in predicting DILI-induced ALF. A validity evaluation of the DSP model using internal validation was performed by bootstrap methods. The AUROC of the DSP model was 0.720 (95% CI:0.682–0.756), and the Brier score used to assess the calibration of the model was 0.044.

An additional independent 402 patients were enrolled in the external validation cohort. The patients were also mainly females (70.1%), the median age of onset was 57.0 years, and the main clinical type was hepatocellular injury (60.9%) (Supplementary Table S1). The median hospitalization time was significantly longer (14.0 days) in patients with the DSP pattern. The rates of ALF (12.7%) and DILI-induced deaths or liver transplantation (3.8%) in the DSP group were higher than that in other groups but without significance (Supplementary Table S2). The AUROC of the DSP model for predicting ALF in the validation cohort was 0.828 (95% CI: 0.788–0.864) with a Brier score of 0.027 (Table 3).

### Optimization of the Double Separate Peak Model by Incorporation of International Normalized Ratio and Alkaline Phosphatase at Drug-Induced Liver Injury Onset

**FIGURE 4:**
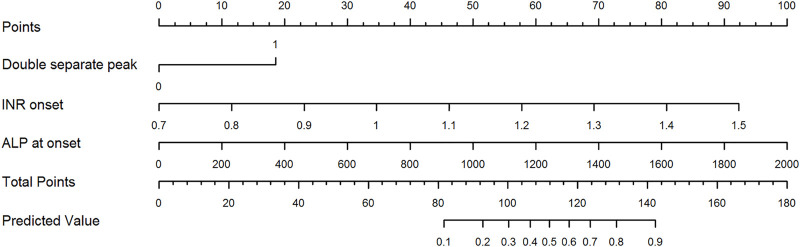
Optimized double separate peak model with INR and ALP at DILI onset. Points are assigned for double separate peak, INR, and ALP at DILI onset using the linear points scale at the top of the figure. The risk of acute liver failure correlating with the total points is on the two linear scales at the bottom of the figure. Abbreviations: ALP, alkaline phosphatase; INR, international normalized ratio.

In light of the role of laboratory parameters other than ALT and TB in the prognostic assessment of DILI, the logistic regression model was used to screen for risk factors for the development of ALF in patients with DILI. When the variables with *p* value <0.05 were included in the multivariate analysis, it was found that INR (OR = 11.8, *p* < 0.001) and ALP at DILI onset (OR = 1.004, *p* = 0.002), complementary with the DSP model (OR = 3.906, *p* = 0.007), were independent risk factors for the development of ALF in patients with DILI, and the logistic regression model was as follows: Logistic (*p*) = 1.347 × DSP pattern + 8.363 × INR + 0.004 × ALP (Figure 4).

Then, we investigated the predictive potency for ALF of the incorporated DSP model with INR and ALP at DILI onset. In the discovery cohort, the AUROC of the optimized model was found to be 0.899 (95% CI: 0.872–0.922), with a sensitivity of 75.0% and a specificity of 90.5%, which was superior to the DSP model alone and the three previous models (Figure 3; Table 3). Internal validation by bootstrap shows that the AUROC was 0.892 with a Brier score of 0.038. The AUROC and Brier scores in the independent validation cohort were 0.958 (95% CI: 0.933–0.975) and 0.022, respectively. Additionally, the AUROC in DILI with NAFLD patients of the optimized model was 0.896 (95% CI: 0.818–0.948), with a sensitivity of 100.0% and a specificity of 81.3%. The value of this new model in the subgroup of DILI with NAFLD has similar predictive potency compared to all DILI patients in terms of ALF.

### Comparison of Pathological Classification According to Alanine Aminotransferase-Total Bilirubin Dynamic Evolution Patterns

**FIGURE 5:**
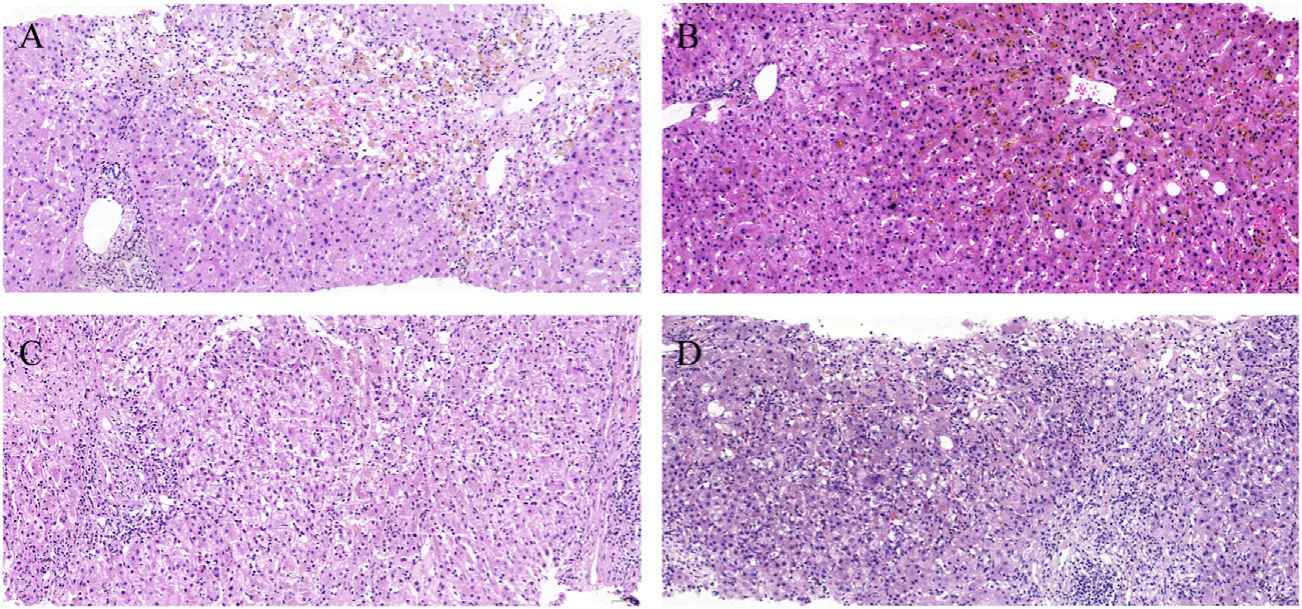
Histological injury patterns. **(A)** Histological acute hepatitis pattern (lobular necroinflammation with cholestasis) mainly correlated with the ALT-mono-peak pattern. **(B)** Histological acute cholestasis pattern (hepatocellular and canalicular cholestasis without obvious necroinflammation) mainly correlated with the TB-mono-peak pattern. **(C)** Histological cholestatic hepatitis pattern (mild to moderate lobular necroinflammation with mild cholestasis) mainly correlated with the ALT and TB double overlap peak pattern. **(D)** Histological cholestatic hepatitis pattern (moderate to severe lobular necroinflammation with moderate cholestasis) mainly correlated with ALT and TB double separate peak patterns. Abbreviations: ALT, alanine aminotransferase; TB, total bilirubin.

A total of 227 patients enrolled in the discovery cohort underwent liver biopsy during hospitalization. Histological injury patterns were shown in Figure 5. The patients with the DSP pattern had predominantly cholestatic hepatitis (75.0%), which was significantly higher than that in the ALT-mono-peak pattern (*p* < 0.05) (Supplementary Table S3). The histological degree of moderate or severe and severe necrosis trended higher in the DSP pattern (29.2%) than in the ALT-mono-peak (11.8), TB-mono-peak (20.0), and DOP (23.2%) patterns.

## Discussion

DILI is the leading cause of ALF in Europe and North America (Stravitz and Lee, 2019) and the third leading cause in China (You et al., 2013; Medina-Caliz et al., 2016). Timely and accurate identification of DILI-induced ALF is the prerequisite for improving the prognosis. Currently, the prognostic models of DILI, such as Hy’s law, nHy’s law, and Robles-Diaz Model, are based on single-point biochemical markers at onset or peak time. In this study, we categorized the dynamic evolution patterns of ALT-TB after DILI onset into four patterns: ALT-mono-peak pattern, TB-mono-peak pattern, DOP pattern, and DSP pattern. The patients with the DSP pattern had significantly more severe disease with a significantly longer hospital stay than other patterns, with an AUROC for predicting DILI-induced ALF of 0.720 (95% CI: 0.682–0.756), a sensitivity of 0.79, and a specificity of 0.66. When we incorporated ALP and INR at DILI onset to the new dynamic model, the AUROC of the optimized model was 0.899 (95% CI: 0.872–0.922) with improvement in both sensitivity and specificity. Furthermore, our results had been validated by an independent external DILI cohort.

The advantages of the new model are the following: first, the most common liver biochemical parameters, namely, ALT and TB, were used—this was simple, not restricted by region, and especially useful in developing countries. The prediction potency can be further improved if incorporated with INR and ALP at DILI onset. Second, we defined the criteria for the dynamic biochemical patterns. DILI patients would usually have the results of a set of their liver biochemical tests before admission. According to these biochemical data, we can easily determine whether ALT or TB is solely or doubly elevated, that is, the mono-peak or double peak patterns. When the liver biochemical tests are checked again during hospitalization, any further increase in TB would implicate the DSP pattern. In the enrolled patients, 86.0% of the DSP pattern was confirmed within 1 week of admission thus implying timely determination of the dynamic patterns without any delay.

In comparison with other predictive models, the DSP model had significantly higher predictive capability of ALF. This is due to it being based not only on the key parameters at onset or peak level but also on the changes within a short period of time, depicting the whole picture along with the natural course of DILI. The kinetics of liver biochemical markers have been used in prediction of treatment response in alcoholic hepatitis, (Rachakonda et al., 2020), chronic hepatitis C (pegylated interferon and ribavirin) (Lee et al., 1998), and acute severe autoimmune hepatitis (Rahim et al., 2020). In the study, we employed them in the prediction of ALF in DILI.

Furthermore, our data showed that DILI patients with or without underlying NAFLD had similar clinical outcomes in terms of ALF and liver-related death/LT. However, the recent guideline suggested that the patients with NASH rather than NAFLD may have an increased risk of severe liver injury and adverse outcome (Regev et al., 2019). Since it is not possible to biopsy all patients with underlying NAFLD to discriminate between NAFL and NASH, the well-accepted non-invasive markers for this discrimination are highly warranted in the field.

Liver pathology determines the severity of liver injury by the lesion characteristic, injury degree, and the regeneration mode, which determines the clinical severity of DILI^22^ (Kleiner, 2018; Kleiner, 2017). Comparison of the histological patterns of DILI according to the various peak patterns revealed that the DSP pattern was associated with more instances of acute cholestatic hepatitis with a higher degree of necrosis and greater cholestasis. The results from the SADRAC database (Robles-Diaz et al., 2014) have shown that the extent of necrosis predicts low survival. Similarly, liver failure and death are associated with more severe necrosis (Robles-Diaz et al., 2014). This histologically accounts for why the DSP pattern is associated with more severe liver injury and hence more likely to be predictive of poorer outcome.

In the study, we proposed, optimized, and validated a novel model which can predict acute ALF after DILI onset with fairly good potency. However, enrolled cases of this study are all hospitalized patients, which are not representative of the entire DILI population. In order to overcome this limitation, we are planning to enroll a prospective DILI cohort to validate the model in a future study as well as the subgroup of DILI with NAFLD (Zhou et al., 2021).

## Conclusion

The dynamic evolution patterns of ALT and TB are correlated with the prognosis of DILI. Patients with the DSP pattern (ALT ≥ 3 × ULN, TB ≥ 2.5 × ULN, with the peak time interval between ALT and TB being ≥3 days) are more likely to progress to ALF. The prediction potency of the model can be further improved by incorporating with INR and ALP at DILI onset so that extra care can be implemented in time for improving the outcomes in patients with DILI.

## Data Availability

The datasets presented in this article are not readily available because the datasets generated and/or analyzed during the current study are not publicly available due to the local policy but are available from the corresponding author on reasonable request. Requests to access the datasets should be directed to zhao_xinyan@ccmu.edu.cn.

## Glossary

AASLD: American Association for the Study of Liver Diseases
ALB: albumin
ALF: acute liver failure
ALP: alkaline phosphatase
ALT: alanine aminotransferase
AST: aspartate aminotransferase
AUROC: area under receiver operating characteristic
BMI: body mass index
CHE: cholinesterase
CK7: cytokeratin 7
CIOMS: Council for International Organizations of Medical Sciences
CI: confidence interval
Cr: creatinine
DILI: drug-induced liver injury
DILIN: drug-induced liver injury network
DOP: ALT and TB double overlap peak pattern
DSP: ALT and TB double separate peak pattern
GGT: gamma glutamyltransferase
HDS: herbal and dietary supplements
H&E: hematoxylin and eosin
HGB: hemoglobin
HIS: hospital management system
INR: international normalized ratio
LT: liver transplantation
NAFLD: non-alcoholic fatty liver disease
nHy’s Law: new Hy’s Law
NSAIDs: non-steroidal anti-inflammatory drugs
PAS-D: periodic acid-Schiff with diastase
PLT: platelets
ROC: receiver operating characteristic
RUCAM: Roussel Uclaf causality assessment method
SADRAC: Swedish Adverse Drug Reactions Advisory Committee
TBA: total bile acid
TB: total bilirubin
ULN: upper limit of normal
WBC: white blood cell
