# Supplementary material for: Alanine Aminotransferase and Bilirubin Dynamic Evolution Pattern as a Novel Model for the Prediction of Acute Liver Failure in Drug-Induced Liver Injury
Source: Front Pharmacol. 2022 Jul 22;13:934467. doi: 10.3389/fphar.2022.934467 (PMC9355525; doi:10.3389/fphar.2022.934467)
Supplement: Supplementary file 1 [file DataSheet1.docx]

**Supplemental Table 1. Comparison of clinical characteristics between discovery and validation cohort**

|  | **Discovery Cohort**  **(n=604)** | **Validation Cohort**  **(n=402)** | ***P* value** |
| --- | --- | --- | --- |
| Female (n, %) | 372 (61.6) | 282 (70.1) | **0.005** |
| Age (years) | 49.0 (40.0, 57.0) | 57.0 (47.0, 64.0) | **<0.001** |
| BMI (kg/m^2^) | 23.3 (21.3, 25.4) | 23.3 (20.9, 25.2) | 0.503 |
| Latency (days) | 27.0 (10.0, 50.0) | 30.0 (10.0, 67.3) | **0.011** |
| R-value onset | 17.0 (6.9, 31.2) | 6.8 (2.6, 13.3) | **<0.001** |
| **Selected liver biochemical tests at DILI onset** | | | |
| ALT (U/L) | 809.5 (321.8, 1262.8) | 303.5 (148.5, 639.0) | **<0.001** |
| AST (U/L) | 546.5 (203.8, 931.0) | 168.0 (84.3, 372.3) | **<0.001** |
| ALP (U/L) | 166.0 (127.0, 226.0) | 153.0 (109.8, 214.0) | **<0.001** |
| GGT (U/L) | 179.0 (100.3, 311.8) | 165.0 (94.5, 282.3) | 0.160 |
| TB (μmol/L) | 113.5 (40.5, 200.1) | 37.6 (17.5, 116.6) | **<0.001** |
| ALB (g/L) | 36.0 (32.0, 38.0) | 36.9 (33.5, 39.7) | **<0.001** |
| TBA (μmol/L) | 80.5 (15.0, 219.8) | 22.4 (7.4, 107.5) | **<0.001** |
| CHOL (mmol/L) | 3.9 (3.2, 4.8) | 4.4 (3.6, 5.2) | **<0.001** |
| TG (mmol/L) | 2.1 (1.4, 3.2) | 1.6 (1.1, 2.2) | **<0.001** |
| INR | 1.0 (0.9, 1.1) | 1.1 (1.0, 1.1) | **<0.001** |
| **Selected liver biochemical tests at their peak time** | | | |
| ALT (U/L) | 829.5 (365.5, 1276.3) | 671.3 (388.8, 1130.1) | **0.028** |
| AST (U/L) | 583.5 (247.3, 965.8) | 445.8 (242.5, 775.0) | **0.002** |
| ALP (U/L) | 185.5 (139.3, 244.0) | 174.5 (126.8, 242.8) | 0.067 |
| GGT (U/L) | 202.0 (117.0, 365.0) | 200.0 (114.5, 331.9) | 0.412 |
| TB (μmol/L) | 145.9 (53.9, 281.6) | 55.1 (21.8, 152.3) | **<0.001** |

Data were presented as median (quartile).

**Abbreviations: DILI,** drug-induced liver injury; **BMI,** Body Mass Index; **ALT,** Alanine aminotransferase; **AST,** Aspartate aminotransferase; **ALP,** Alkaline phosphatase; **GGT,** Glutamyltransferase; **TB,** Total bilirubin; **ALB,** Albumin; **TBA,** Total bile acid; **CHOL,** Cholesterol; **TG,** Triglycerides; **INR,** International Normalized Ratio.

**Supplemental Table 2. Comparison of the demographic and liver biochemical parameters among four ALT-TB dynamic evolution patterns in DILI from validation cohort**

| **Validation cohort** | **Total**  **(n=402)** | **ALT-mono-peak (n=183)** | **TB-mono-peak (n=16)** | **ALT and TB double overlap peak (n=124)** | **ALT and TB double separate peak (n= 79)** | ***P* value** |
| --- | --- | --- | --- | --- | --- | --- |
| Female (n, %) | 282 (70.1) | 142 (77.6) | 10 (62.3) | 78 (62.9%) | 52 (65.8%) | **0.028**^●^ |
| Age (years) | 57.0 (47.0, 64.0) | 57.0 (48.0, 63.0) | 59.5 (48.8, 63.8) | 56.0 (45.0, 64.8) | 58.0 (48.0, 66.0) | 0.823 |
| BMI (kg/m^2^) | 23.4 (20.8, 25.2) | 23.5 (21.3, 25.4) | 21.8 (19.7, 24.6) | 23.2 (20.4, 25.0) | 23.4 (21.1, 25.0) | 0.425 |
| Latency (days) | 30.0 (10.0, 67.3) | 31.0 (14.0, 76.3) | 17.0 (4.5, 62.5) | 26.0 (10.0, 70.3) | 25.0 (8.0, 55.0) | 0.231 |
| Duration of hospitalization (days), n (%) | 11.0 (8.0, 14.0) | 9.0 (7.0, 12.0) | 12.0 (9.3, 20.5) | 11.0 (8.0, 14.0) | 14.0 (12.0, 18.0) | **<0.001**^●★^**^✝^** |
| R-value onset | 6.8 (2.6, 13.3) | 7.3 (3.8, 13.2) | 0.7 (0.4, 1.0) | 7.7 (3.2, 14.7) | 7.4 (2.2, 13.9) | **<0.001**^◆▲▼^ |
| **Injury patterns, n (%)** | | | | | | **<0.001** |
| Hepatocellular | 245 (60.9) | 122 (66.7) | 0 (0.0) | 78 (62.9) | 45 (57.0) | ^◆▲▼^ |
| Cholestatic | 78 (19.4%) | 21 (11.5) | 16 (100.0) | 23 (18.5) | 18 (22.8) | ^◆▲▼^ |
| Mixed | 79 (19.7%) | 40 (21.9) | 0 (0.0) | 23 (18.5) | 16 (20.3) |  |
| **The culprit drug(s), n (%)** | | | | | | **0.028** |
| HDS | 191 (47.5) | 79 (43.2) | 3 (18.8) | 62 (50.0) | 43 (54.4) |  |
| Drugs | 100 (24.9) | 40 (21.6) | 10 (62.5) | 32 (25.8) | 22 (27.8) | ^◆▲▼^ |
| HDS +Drugs | 111 (27.6) | 64 (35.1) | 3 (18.8) | 30 (24.2) | 14 (17.7) | ^★^ |
| **Selected liver biochemical tests at DILI onset** | | | | | | |
| ALT (U/L) | 303.5 (148.5, 639.0) | 282.0 (151.0, 574.0) | 41.5 (28.5, 57.9) | 376.0 (231.5, 721.8) | 340.0 (144.0, 823.0) | **<0.001**^◆▲▼^ |
| AST (U/L) | 168.0 (84.3, 372.3) | 148.0 (80.4, 303.0) | 52.3 (33.1, 72.3) | 190.5 (111.2, 483.0) | 275.0 (106.0, 658.8) | **<0.001**^◆●★▲▼^ |
| ALP (U/L) | 153.0 (109.8, 214.0) | 126.0 (98.0, 178.0) | 196.5 (158.0, 279.3) | 162.5 (115.0, 241.5) | 179.0 (119.0, 232.0) | **<0.001**^◆●★^ |
| GGT (U/L) | 165.0 (94.5, 282.3) | 150.0 (82.0, 251.0) | 85.0 (42.3, 195.1) | 184.0 (123.0, 294.5.0) | 213.0 (111.0, 365.0) | **0.001**^★▲▼^ |
| TB (μmol/L) | 37.6 (17.5, 116.6) | 17.0 (12.1, 26.7) | 211.2 (96.5, 285.2) | 78.6 (38.2, 154.5) | 157.1 (69.0, 273.5) | **<0.001**^◆●★^**^✝^** |
| ALB (g/L) | 36.9 (33.5, 39.7) | 38.1 (35.5, 40.3) | 34.3 (31.3, 37.1) | 35.6 (32.9, 39.0) | 35.3 (30.7, 39.5) | **<0.001**^◆●★^ |
| CHE (KU/L) | 6.6 (5.1, 7.8) | 7.4 (6.3, 8.5) | 5.7 (3.5, 6.9) | 5.6 (4.6, 6.9) | 5.6 (4.5, 6.9) | **<0.001**^◆●★^ |
| TBA (μmol/L) | 22.4 (7.4, 107.5) | 8.9 (4.4, 20.5) | 148.5 (104.9, 256.0) | 37.3 (13.8, 121.0) | 130.5 (57.3, 216.1) | **<0.001**^◆●★^**^✝^** |
| CHOL (mmol/L) | 4.4 (3.6, 5.2) | 4.5 (3.9, 5.1) | 5.4 (3.6, 7.5) | 4.1 (3.4, 5.1) | 4.0 (3.3, 5.4) | **0.048** |
| TG (mmol/L) | 1.6 (1.1, 2.2) | 1.2 (1.0, 1.6) | 2.8 (2.1, 4.5) | 1.7 (1.2, 2.3) | 2.2 (1.6, 3.5) | **<0.001**^◆●★▲^ |
| INR | 1.0 (1.0, 1.1) | 1.0 (1.0, 1.1) | 1.0 (1.0, 1.1) | 1.1 (1.0, 1.2) | 1.1 (1.0, 1.2) | **<0.001**^●★^ |
| **Selected liver biochemical tests at their peak time** | | | | | | |
| ALT (U/L) | 671.3 (388.8, 1130.1) | 623.0 (385.3, 958.7) | 44.5 (30.3, 60.9) | 900.5 (562.3, 1342.5) | 720.0 (409.0, 1298.0) | **<0.001**^◆●▲▼^ |
| AST (U/L) | 445.8 (242.5, 775.0) | 315.0 (211.0, 551.5) | 51.8 (39.9, 77.0) | 685.5 (396.5, 945.0) | 676.0 (312.0, 1071.0) | **<0.001**^◆●★▲▼^ |
| ALP (U/L) | 174.5 (126.8, 242.8) | 150.0 (106.0, 217.0) | 206.0 (179.3, 243.0) | 183.0 (142.0, 313.0) | 193.0 (150.0, 253.0) | **<0.001**^◆●★^ |
| GGT (U/L) | 200.0 (114.5, 331.9) | 176.0 (92.0, 258.0) | 109.6 (60.3, 247.0) | 231.0 (141.0, 372.9) | 250.4 (171.0, 380.0) | **<0.001**^●★▲▼^ |
| TB (μmol/L) | 55.1 (21.8, 152.3) | 20.0 (14.5, 28.5) | 285.5 (163.3, 344.3) | 113.6 (78.3. 203.6) | 152.7 (83.0, 244.2) | **<0.001**^◆●★^ |
| **Severity, n (%)** | | | | | | **<0.001** |
| Mild | 192 (47.8) | 183 (100.0) | 1 (6.3) | 5 (4.0) | 3 (3.8) | ^◆●★^ |
| Moderate | 36 (9.0) | 0 (0.0) | 0 (0.0) | 29 (23.4) | 7 (8.9) | ^●★^**^✝^** |
| Severe | 162 (40.3) | 0 (0.0) | 15 (93.8) | 88 (71.0) | 59 (74.7) | ^◆●★^ |
| ALF/Fatal | 12 (3.0) | 0 (0.0) | 0 (0.0) | 2 (1.6) | 10 (12.7) | **^✝^** |
| **Outcomes, n (%)** | | | | | |  |
| Acute liver failure | 12 (3.0) | 0 (0.0) | 0 (0.0) | 2 (1.6) | 10 (12.7) | **<0.001**^★^ |
| Liver-related Death/LT | 3 (0.7) | 0 (0.0) | 0 (0.0) | 0 (0.0) | 3 (3.8) | **0.006**^★^ |

Data were presented as median (quartile).

**Abbreviations: DILI,** drug-induced liver injury; **BMI,** Body Mass Index; **HDS,** Herbal and dietary supplements; **ALT,** Alanine aminotransferase; **AST,** Aspartate aminotransferase; **ALP,** Alkaline phosphatase; **GGT,** Glutamyltransferase; **TB,** Total bilirubin; **ALB,** Albumin; **CHE,** Cholinesterase; **TBA,** Total bile acid; **CHOL,** Cholesterol; **TG,** Triglycerides; **INR,** International Normalized Ratio; **LT,** Liver transplantation.

^◆^There is a statistical difference between ALT-mono-peak and TB-mono-peak;

^●^There is a statistical difference between ALT-mono-peak and double overlap peak;

^★^There is a statistical difference between ALT-mono-peak and double separate peak;

^▲^There is a statistical difference between TB-mono-peak and double overlap peak;

^▼^There is a statistical difference between TB-mono-peak and double separate peak;

**^✝^**There is a statistical difference between double overlap peak and double separate peak.

**Supplemental Table 3. Comparison of histological characteristics among four ALT-TB dynamic evolution patterns in DILI**

|  | **Total (n=227)** | **ALT-mono-peak (n=51)** | **TB-mono-peak (n=5)** | **ALT and TB double overlap peak (n=99)** | **ALT and TB double separate peak (n=72)** | **P Value** |
| --- | --- | --- | --- | --- | --- | --- |
| **Histological injury patterns, n (%)** | | | | | | **<0.001** |
| Acute hepatitis | 80 (35.2) | 30 (58.8) | 0 (0.0) | 34 (34.3) | 16 (22.2) | ^●★^ |
| Chronic hepatitis | 0 (0.0) | 0 (0.0) | 0 (0.0) | 0 (0.0) | 0 (0.0) |  |
| Acute cholestasis | 4 (1.8) | 0 (0.0) | 0 (0.0) | 2 (2.0) | 2 (2.8) |  |
| Chronic cholestasis | 0 (0.0) | 0 (0.0) | 0 (0.0) | 0 (0.0) | 0 (0.0) |  |
| Cholestatic hepatitis | 143 (63.0) | 21 (41.2%) | 5 (100.0%) | 63 (63.6%) | 54 (75.0%) | ^★^ |
| **Severity of pathological necrosis, n (%)** | | | | | | **0.084** |
| mild | 77 (33.9) | 24 (47.1) | 1 (20.0) | 31 (31.3) | 21 (29.2) |  |
| Moderate | 99 (43.6) | 21 (41.2) | 3 (60.0) | 45 (45.5) | 30 (41.7) |  |
| Moderate or severe | 47 (20.7) | 6 (11.8) | 1 (20.0) | 23 (23.2) | 17 (23.6) |  |
| Severe | 4 (1.8) | 0 (0.0) | 0 (0.0) | 0 (0.0) | 4 (5.6) |  |

**Abbreviations: DILI,** drug-induced liver injury; **ALT,** Alanine aminotransferase; **TB,** Total bilirubin.

^●^There is a statistical difference between ALT-mono-peak and double overlap peak;

^★^There is a statistical difference between ALT-mono-peak and double separate peak.

**Supplemental Table 4. Comparison of demographic and laboratory data at DILI onset between ALF group and non-ALF group.**

|  | **Total (n=604)** | **ALF (n=28)** | **non-ALF without LT (n=576)** | **P value** |
| --- | --- | --- | --- | --- |
| Female (n, %) | 372 (61.6) | 22 (78.6) | 350 (60.8) | 0.059 |
| Age (yr) | 49.0 (40.0, 57.0) | 51.0 (42.8, 56.0) | 49.0 (40.0, 57.0) | 0.384 |
| BMI (kg/m^2^) | 23.3 (21.3, 25.4) | 23.2 (21.2, 25.4) | 23.3 (21.3, 25.4) | 0.776 |
| Implicated drugs (n, %) |  |  |  | 0.246 |
| HDS | 302 (50.0) | 17 (60.7) | 285 (49.5) |  |
| non-HDS | 302 (50.0) | 11 (39.3) | 291 (50.5) |  |
| Latency (n, %) | 27.0 (10.0, 50.0) | 14.0 (7.0, 30.0) | 30.0 (10.0, 60.0) | **0.010** |
| R-value onset | 17.0 (6.9, 31.2) | 16.9 (8.2, 24.1) | 17.0 (6.7, 32.0) | 0.576 |
| **Selected liver biochemical tests at DILI onset** | | | | |
| ALT (U/L) | 809.5 (321.7, 1262.8) | 794.0 (362.5, 1245.8) | 810.5 (319.5, 1262.8) | 0.913 |
| AST (U/L) | 546.5 (203.8, 931.0) | 649.0 (230.5, 1006.5) | 538.5 (200.8, 930.5) | 0.292 |
| ALP (U/L) | 166.0 (127.0, 226.0) | 210.0 (146.5, 255.8) | 165.0 (126.0, 223.8) | 0.061 |
| GGT (U/L) | 179.0 (100.3, 311.8) | 175.5 (92.3, 318.5) | 179.0 (100.3, 311.8) | 0.807 |
| TB (μmol/L) | 113.5 (40.5, 200.1) | 190.9 (100.7, 309.0) | 110.0 (38.0, 193.8) | **0.001** |
| ALB (g/L) | 36.0 (32.0, 38.0) | 31.5 (30.0, 35.0) | 36.0 (33.0, 39.0) | **<0.001** |
| TBA (μmol/L) | 80.5 (15.0, 219.8) | 219.0 (108.3, 300.0) | 73.5 (13.3, 209.8) | **<0.001** |
| INR | 1.0 (0.9, 1.1) | 1.2 (1.1, 1.4) | 1.0 (0.9, 1.1) | **<0.001** |

Data were presented as median (quartile).

**Abbreviations: ALB,** Albumin; **ALF,** acute liver failure; **ALP,** alkaline phosphatase; **ALT,** alanine amino transferase; **AST,** aspartate amino transferase; **BMI,** Body Mass Index; **GGT,** gamma glutamyl transferase; **HDS,** Herbal and dietary supplements; **INR,** international normalized ratio; **TBA,** total bile acid; TB, total bilirubin.

**Supplemental Table 5. Comparison of demographic and laboratory data at DILI onset** **between DILI with NAFLD and DILI without NAFLD group.**

|  | **Total (n=604)** | **DILI with NAFLD (n=99)** | **DILI without NAFLD (n=576)** | **P value** |
| --- | --- | --- | --- | --- |
| Female (n, %) | 372 (61.6) | 52 (52.5) | 320 (63.4) | **0.043** |
| Age (yr) | 49.0 (40.0, 57.0) | 51.0 (40.0, 60.0) | 49.0 (40.0, 57.0) | 0.196 |
| BMI (kg/m^2^) | 23.3 (21.3, 25.4) | 24.8 (23.1, 26.5) | 22.9 (21.2, 25.0) | **<0.001** |
| Implicated drugs (n, %) |  |  |  | **0.011** |
| HDS | 302 (50.0) | 38 (38.4) | 264 (52.3) |  |
| non-HDS | 302 (50.0) | 61 (61.6) | 241 (47.7) |  |
| Latency (n, %) | 27.0 (10.0, 50.0) | 20.0 (5.0, 60.0) | 30.0 (10.0, 50.0) | 0.172 |
| R-value onset | 17.0 (6.9, 31.2) | 18.6 (9.9, 36.3) | 16.5 (6.1, 30.5) | 0.105 |
| **Selected liver biochemical tests at DILI onset** | | | | |
| ALT (U/L) | 809.5 (321.7, 1262.8) | 803.0 (414.0, 1305.0) | 811.0 (312.0, 1254.0) | 0.255 |
| AST (U/L) | 546.5 (203.8, 931.0) | 449.0 (200.0, 857.0) | 576.0 (204.5, 947.5) | 0.495 |
| ALP (U/L) | 166.0 (127.0, 226.0) | 162.0 (118.0, 204.0) | 167.0 (127.5, 227.5) | 0.159 |
| GGT (U/L) | 179.0 (100.3, 311.8) | 221.0 (118.0, 401.0) | 170.0 (100.0, 297.0) | **0.020** |
| TB (μmol/L) | 113.5 (40.5, 200.1) | 84.3 (22.8, 170.0) | 120.0 (47.4, 204.8) | **0.002** |
| ALB (g/L) | 36.0 (32.0, 38.0) | 37.0 (32.0, 39.0) | 36.0 (32.0, 38.0) | 0.212 |
| TBA (μmol/L) | 80.5 (15.0, 219.8) | 44.0 (8.0, 164.0) | 89.0 (16.0, 232.5) | **0.002** |
| INR | 1.0 (0.9, 1.1) | 1.0 (0.9, 1.1) | 1.0 (0.9, 1.1) | 0.703 |
| **Outcomes, n (%)** | | | | |
| Acute liver failure | 28 (4.6) | 3 (3.0) | 25 (5.0) | 0.406 |
| Liver-related Death/LT | 13 (2.2) | 2 (2.0) | 11 (2.2) | 0.921 |

Data were presented as median (quartile).

**Abbreviations: ALB,** Albumin; **ALF,** acute liver failure; **ALP,** alkaline phosphatase; **ALT,** alanine amino transferase; **AST,** aspartate amino transferase; **BMI,** Body Mass Index; **GGT,** gamma glutamyl transferase; **HDS,** Herbal and dietary supplements; **INR,** international normalized ratio; **LT,** liver transplantation; **TBA,** total bile acid; **TB,** total bilirubin.
